# Vessel wall magnetic resonance and arterial spin labelling imaging in the management of presumed inflammatory intracranial arterial vasculopathy

**DOI:** 10.1093/braincomms/fcac157

**Published:** 2022-06-20

**Authors:** L A Benjamin, E Lim, M Sokolska, J Markus, T Zaletel, V Aggarwal, R Luder, E Sanchez, K Brown, R Sofat, A Singh, C Houlihan, E Nastouli, N Losseff, D J Werring, M M Brown, J C Mason, R J Simister, H R Jäger

**Affiliations:** Comprehensive Stroke Service, National Hospital for Neurology and Neurosurgery, University College London Hospitals NHS Foundation Trust, Queen Square, Box 16, London WC1N 3BG, UK; Laboratory of Molecular and Cell Biology, UCL, Gower St, Kings Cross, London WC1E 6BT, UK; Stroke Research Centre, UCL Queen Square Institute of Neurology, University College London, London WC1B 5EH, UK; University of Liverpool, Brain Infections Group, Liverpool, Merseyside, L69 7BE, UK; Department of Imaging, University College London Hospitals NHS foundation trust, London, NW1 2PG, UK; Department of Medical Physics and Biomedical Engineering, University College London Hospitals NHS Foundation Trust, London, NW1 2PG, UK; Department of Imaging, University College London Hospitals NHS foundation trust, London, NW1 2PG, UK; Department of Medicine, University of Cambridge, Cambridge, CB2 1TN, UK; Comprehensive Stroke Service, National Hospital for Neurology and Neurosurgery, University College London Hospitals NHS Foundation Trust, Queen Square, Box 16, London WC1N 3BG, UK; Department of Medicine, North Middlesex University Hospital, London, N18 1QX, UK; Department of clinical virology, University College London Hospitals NHS Foundation Trust, London, NW1 2PG, UK; Department of Virology, UK Health Security Agency, London, NW9 5EQ, UK; Department of Pharmacology and Therapeutics, University of Liverpool, Liverpool L69 7BE, UK; Health Data Research, London, NW1 2BE, UK; Department of Medicine, Royal Free Hospital Foundation Trust, London, NW3 2QG, UK; Department of clinical virology, University College London Hospitals NHS Foundation Trust, London, NW1 2PG, UK; Department of clinical virology, University College London Hospitals NHS Foundation Trust, London, NW1 2PG, UK; Crick Institute, London, NW1 1AT, UK; Comprehensive Stroke Service, National Hospital for Neurology and Neurosurgery, University College London Hospitals NHS Foundation Trust, Queen Square, Box 16, London WC1N 3BG, UK; Comprehensive Stroke Service, National Hospital for Neurology and Neurosurgery, University College London Hospitals NHS Foundation Trust, Queen Square, Box 16, London WC1N 3BG, UK; Stroke Research Centre, UCL Queen Square Institute of Neurology, University College London, London WC1B 5EH, UK; Stroke Research Centre, UCL Queen Square Institute of Neurology, University College London, London WC1B 5EH, UK; Department of Medicine, Hammersmith Hospital, London, W12 0HS, UK; National Heart and Lung Institute, Imperial College London, London, SW3 6LY, UK; Comprehensive Stroke Service, National Hospital for Neurology and Neurosurgery, University College London Hospitals NHS Foundation Trust, Queen Square, Box 16, London WC1N 3BG, UK; Stroke Research Centre, UCL Queen Square Institute of Neurology, University College London, London WC1B 5EH, UK; Stroke Research Centre, UCL Queen Square Institute of Neurology, University College London, London WC1B 5EH, UK; Department of Imaging, University College London Hospitals NHS foundation trust, London, NW1 2PG, UK; Neuroradiological Academic Unit, UCL Queen Square Institute of Neurology, University College London, London, WC1N 3BG, UK

**Keywords:** cerebral vasculitis, neuroinflammation, stroke, vessel wall MR, ASL

## Abstract

Optimal criteria for diagnosing and monitoring response to treatment for infectious and inflammatory medium–large vessel intracranial vasculitis presenting with stroke are lacking. We integrated intracranial vessel wall MRI with arterial spin labelling into our routine clinical stroke pathway to detect presumed inflammatory intracranial arterial vasculopathy, and monitor disease activity, in patients with clinical stroke syndromes. We used predefined standardized radiological criteria to define vessel wall enhancement, and all imaging findings were rated blinded to clinical details. Between 2017 and 2018, stroke or transient ischaemic attack patients were first screened in our vascular radiology meeting and followed up in a dedicated specialist stroke clinic if a diagnosis of medium–large inflammatory intracranial arterial vasculopathy was radiologically confirmed. Treatment was determined and monitored by a multi-disciplinary team. In this case series, 11 patients were managed in this period from the cohort of young stroke presenters (<55 years). The median age was 36 years (interquartile range: 33,50), of which 8 of 11 (73%) were female. Two of 11 (18%) had herpes virus infection confirmed by viral nucleic acid in the cerebrospinal fluid. We showed improvement in cerebral perfusion at 1 year using an arterial spin labelling sequence in patients taking immunosuppressive therapy for >4 weeks compared with those not receiving therapy [6 (100%) versus 2 (40%) *P* = 0.026]. Our findings demonstrate the potential utility of vessel wall magnetic resonance with arterial spin labelling imaging in detecting and monitoring medium–large inflammatory intracranial arterial vasculopathy activity for patients presenting with stroke symptoms, limiting the need to progress to brain biopsy. Further systematic studies in unselected populations of stroke patients are needed to confirm our findings and establish the prevalence of medium–large artery wall inflammation.

## Teaser

Benjamin *et al.* demonstrated, in a longitudinal case series of stroke patients, the utility of intracranial vessel wall magnetic resonance imaging, combined with arterial spin labelling in the detection and monitoring of patients with medium–large intracranial non-atherosclerotic inflammatory vasculopathy; providing the foundation to formally evaluate the burden and support vital trials.

## Graphical Abstract

**Graphical abstract:**
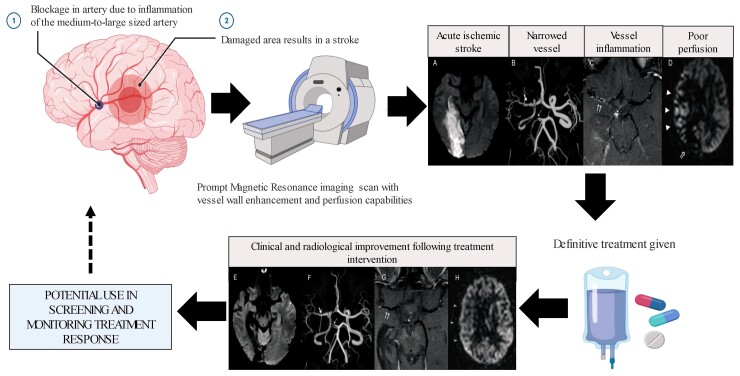

## Introduction

Cryptogenic stroke not caused by large artery atherosclerosis, cardioembolism or small vessel occlusion, is common and accounts for ∼one-third of ischaemic stroke presentations.^1^ Very little is known to what extent uncommon but potentially treatable aetiologies, such as medium–large vessel intracranial vasculitis, contribute to this substantial patient group but, the wider availability of arterial imaging at hospital presentation is increasingly identifying patients with intracranial artery pathology without a cause.

Vasculitis is a term reserved for when there is histopathological confirmation with characteristic granulomatous, lymphocytic or necrotising vessel inflammation alongside vessel wall damage. In the absence of histology, we use the term vasculopathy.^2^ The causes of medium–large vessel intracranial vasculitis are multifactorial, including infection, autoimmune, neoplastic, metabolic and genetic conditions.^3^ It can be primary (i.e. limited to the brain) or secondary (i.e. involving the systemic circulation).^3^ For the former, primary central nervous system vasculitis (PCNSV) is the recognized term and classically affects small vessels.^3^ The systematic testing for medium–large vessel intracranial vasculitis occurs only rarely through stroke services, and the diagnosis is likely to be frequently missed.^4^ The paradigm shift of ‘front door’ stroke management rightly focuses on hyperacute interventions, such as intravenous thrombolysis and mechanical thrombectomy for ischaemic stroke, which save lives and disability.^5^ Dual antiplatelet treatment for minor stroke and high risk transient ischaemic attack (TIA) are now commonly introduced early after presentation to minimize recurrence. These care pathways can delay prompt screening for treatable causes of ischaemic stroke, including medium–large vessel intracranial vasculitis possibly triggered by infections. Lumbar puncture (LP), which is important for diagnosis, can be significantly delayed because of prior or acute antiplatelet use, specifically with clopidogrel.^6^

Cerebral biopsy remains the gold standard diagnostic tool in the diagnosis of PCNSV, but its sensitivity is low and has very limited evidence base for exclusively medium–large vessel inflammation presentations.^7^ Though the benefit of ^18^F-FDG PET-CT/MR for detection and disease activity surveillance has been reported for large vessel systemic vasculitis; its utility is not established in the central nervous system.^8^ For the purpose of this article, we reserved the term vasculitis for histologically proven cases and presumed inflammatory intracranial arterial vasculopathy when this was not available.

The perceived rarity of intracranial vasculitis as a cause of a first stroke presentation, attributable to medium–large arterial disease, is that specific investigations for vasculitis are rarely performed or considered only after repeated unexplained stroke events. In addition, uncertainty as to the optimal investigation pathway in suspected presentations can further limit case assessment to a level that justifies the use of immunosuppressive therapies with potentially significant side effects.

Intracranial vessel wall MR is an emerging technology that might help mitigate these limitations in diagnosing and monitoring response to treatment in patients with presumed inflammatory intracranial arterial vasculopathy. It involves high-resolution fat-saturated T_1_-weighted black blood images, pre- and post-contrast which allows visualization of the vessel wall and detection of inflammatory changes within it.^9,10^ Some studies have already demonstrated the diagnostic capabilities of intracranial vessel wall MRI in inflammatory vasculopathies.^11–16^ Arterial spin labelling (ASL) MR perfusion imaging is a non-invasive technique to assess cerebral blood flow, which is currently transitioning from research into clinical applications.^17^ We report our experience in establishing an optimised protocol that combines intracranial vessel wall MRI with ASL for patients with suspected inflammatory intracranial arterial vasculopathy and its integration into our clinical stroke service. As a result, we have been able to detect presumed inflammatory intracranial arterial vasculopathy associated with various causes and measure its haemodynamic impact. Our observations expand on the spectrum of treatable conditions in acute stroke and highlight the importance of diagnosis and management of presumed inflammatory intracranial arterial vasculopathy.

## Subjects/materials and methods

### Selection criteria

We identified all patients managed in our dedicated multi-disciplinary stroke clinic with a presumed inflammatory intracranial arterial vasculopathy diagnosis presenting between 2017 and 2018 to the hyperacute stroke unit and TIA services at University College London Hospitals (UCLHs) NHS Foundation Trust. The stroke and TIA services manage ∼1000 ischaemic strokes and 500 TIA per year, and UCLH is the primary centre for assessment of all new stroke events in the North Central London region (population 1.2 million). Approximately 20% will be <55 years old. Routine inclusion of arterial imaging in all first-line assessments has been standard practice since 2010. All patients had presented with a stroke event judged by the service vascular review meeting to be secondary to medium–large vessel intracranial artery pathology and no evidence of conventional causation (i.e. no evidence of conventional atherosclerosis, cardiac embolism or pattern suggestive of a reversible vasoconstriction syndrome) and further evaluated in the specialist service. We included the subset of patients aged ≤55years for this review to minimize any confounding effect of coincident background atherosclerosis. Each patient underwent baseline intracranial vessel wall MRI with ASL and had follow-up imaging at 6 months and 1 year as part of their routine clinical care.^9^

### MRI protocol

Intracranial vessel wall MRI with ASL was performed on a 3 T MRI Philips Achieva system (Philips Healthcare, Best, the Netherlands) with a 32-channel head coil. Brain imaging included diffusion-weighted imaging, susceptibility-weighted imaging, fluid-attenuated inversion recovery and pseudo-continuous ASL with a post-labelling delay of 2 s. Intracranial vessel wall MRI consisted of high-resolution time of flight (ToF) and black blood imaging: fat-suppressed turbo spin echo (FS-TSE) with a voxel size of 0.4 × 0.4 and 2 mm thickness. FS-TSE sequences were performed in coronal plane pre-contrast, followed by coronal and axial planes after administration of contrast. In some cases, sagittal FS-TSE or TSE with quadruple inversion recovery preparation pulses was performed to assess petrous and cavernous portions of the internal carotid artery (ICA).

### Imaging analysis

Two neuroradiologists independently assessed the MRI findings, blinded to the clinical history. H.R.J. has 8 years of experience in vessel wall imaging. He is one of the pioneers of its clinical implementation in the UK and is a nationally and internationally renowned speaker and teacher on this subject area. E.L. was trained by H.R.J. and had 2 years of experience in vessel wall imaging. They evaluated disease activity with regards to (i) vessel wall enhancement, looking for the characteristic tramline or circumferential enhancement seen in vasculitis, (ii) degree of stenosis, (iii) cerebral perfusion at baseline, 6 months and 1 year. All 1-year measures were compared with baseline scans and defined as 1 = improvement and 2 = no change/progression. The vasa vasorum can show tramline enhancement; in the population group assessed, this is usually present in the petrous and cavernous portions of the ICA. We therefore only considered an enhancement of the petrous ICA pathological when it was associated with marked wall thickening and clearly more marked than the physiological enhancement of the vasa vasorum on the contralateral side. For the analysis of perfusion-weighted ASL images, we adopted a previously described method, which assesses the presence and severity of arterial transit artefacts (ATAs) in anatomical regions based on a modified ASPECT score for cortical regions.^18^ ATAs occur when the labelled spins have not fully reached the cortex and are still in leptomeningeal vessels. This gives rise to serpiginous high signal areas on the brain's surface and suggests a delay in perfusion. We used the previously described rating of perfusion on ASL images: 0, no or minimal ASL signal; 1, moderate ASL signal with ATA; 2, high ASL signal with ATA; and 3, normal perfusion without ATAs. With 10 regions being assessed, this cumulated to a maximum score of 30 for a normally perfused hemisphere. Using this ASL perfusion score, we were able to quantify the interval changes on ASL images in the different treatment groups.^18,19^ The *k* statistic was calculated. For disagreements, a consensus was reached.

### Inter-reader agreement

There was no difference between the two readers in identifying crude changes in enhancement, stenosis, and perfusion. A difference was encountered with the ASPECT grading for reading (*k* = 0.95); an agreement was reached after a joint review of the case.

### Classification of presumed inflammatory intracranial arterial vasculopathy affecting medium–large arteries

To gain a better understanding of the heterogeneous nature of this group, we classified those with intracranial arterial vessel wall MR scans suggestive of an inflammatory aetiology into three groups as follows.

1. Infective: CSF positive for nucleic acid, antigen, intrathecal antibody, or culture-positive to a specific infection.
2. Radiological evidence of inflammation with supporting evidence from additional testing (Inflam+): CSF negative for nucleic acid, antigen, intrathecal antibody, or culture-positive to a specific infection, and CSF pleocytosis (≥5 cells/mm3), or CSF protein 2-fold above the upper limit of normal (to take account of elevation due to a stroke), or elevated CSF IgG index, or whole-body ^18^F-FDG PET arterial reactivity/avidity.
3. Radiological evidence of inflammation with no supporting evidence from additional testing (Inflam-): CSF negative for nucleic acid, antigen, intrathecal antibody, or culture-positive to a specific infection, with no CSF pleocytosis (≥5 cells/mm3), no CSF protein rise >2-fold above the upper limit of normal, no CSF IgG index elevation, and no evidence of whole-body ^18^F-FDG PET arterial reactivity/avidity.

### Management of the patients

Patients with radiological confirmation of presumed inflammatory intracranial arterial vasculopathy affecting medium–large arteries using vessel wall MRI with ASL were referred to a multi-disciplinary team (MDT) for a discussion about diagnosis and management. The MDT included two stroke neurologists (one with infectious disease expertise), a neuroradiologist, and a rheumatologist with expertise in vasculitis. Confirmed circumferential or tramline vessel wall enhancement on intracranial vessel wall MR was considered characteristic of vasculitis, therefore presumed to have an inflammatory intracranial arterial vasculopathy. Patients with this finding were selected for a further battery of tests by the MDT.^9^ This included cerebrospinal fluid (CSF) examination to look for evidence of inflammation and infection by PCR or intrathecal antibody synthesis and autoimmune, metabolic and infection screen blood tests. Thrombophilia screen was requested when brain imaging was consistent with multi-focal infarcts in the absence of a cardioembolic source and/or evidence of systemic thrombosis or clotting abnormalities.^18^F-FDG PET was considered in all patients to look for evidence of systemic vasculitis and a possible systemic biopsy target. Among those with brain biopsy and confirmed inflammatory intracranial arterial vasculopathy on imaging, unbiased infection screening strategies such as metagenomic analysis was considered when there was a high degree of suspicion of an infectious aetiology and a negative comprehensive infection screen. For example, a progressive history in the context of immunosuppression was considered to be suspicious of infection. All patients also had standard stroke workup, including cardiac echocardiogram, 72 h ECG monitoring and when imaging was consistent with an embolic source, a bubble echocardiogram and transesophageal echocardiogram were requested. The patient’s progress, with or without treatment, was monitored and fed back to the MDT to guide further management. In the absence of definitive evidence for the management of presumed inflammatory intracranial arterial vasculopathy, treatment choices were practical, individualized after discussion and drawn from the experience of managing large vessel systemic vasculitis (e.g. giant cell and Takayasu Arteritis) with use of drugs such as methotrexate, cyclophosphamide, mycophenolate mofetil and Tocilizumab (Supplementary Fig. 1). If treatment was delayed, and monitoring indicated active disease (i.e. progressive vasculopathy, new clinical or radiological event), treatment was often advocated. The clinical history, vascular risk factors and treatment history were recorded and summarized.

### Statistical analysis

Continuous variables were summarized using means and medians and compared using student independent-samples *t*-test or Mann–Whitney U test as appropriate. Categorical data were represented as percentages and compared using Fisher exact. Individuals with missing data were excluded from that analysis. We did not adjust for all covariates because of the small sample size. We considered a two-sided *P* value <0.05 to indicate statistical significance. We used statistical software (Stata Statistical Software, version 15.1; StataCorp, College Station, Tex) for all analyses.

### Ethics

Standard protocol approvals and patient consent UCLH stroke service routine collection of clinical data are approved by the UCLH Governance Review Board as a continuous service evaluation of a comprehensive care programme (service evaluation 5-201929-SE); for this reason, informed patient consent was not routinely required, but individual patient consent for the expanded cases was obtained.

### Data availability

The data are available upon reasonable request and ethical approval.

## Results

### Patient characteristics

Eleven patients were included in this case series presenting to the service between January 2017 and June 2018. They were each followed up for 1 year. The median age was 36 years [interquartile range (IQR): 33,50], 8 of 11 (73%) were women. None of the cases had evidence of brain ^18^F-FDG PET activity, and CSF pleocytosis was infrequent [2/9 (22%)]. All patients were alive at one year. The patient characteristics are described as a summary in Table 1 and in detail in Table 2.

**Table 1:** Summary of patient characteristics with medium–large vessel intracranial vasculitis

		Infective (*n* = 2)	Inflam+ (*n* = 3)	Inflam- (*n* = 6)
Female n (%)	8 (73)	2 (100)	3 (100)	3 (50)
Median age years (IQR)	36 (33,50)	30	41	45
Type of inflammatory vasculopathy n (%)		—	—	—
*Infective*	2 (18)			
*Inflam+*	3 (27)			
*Inflam-*	6 (55)			
PET scan—evidence of brain avidity *n* (%)	0	—	—	—
PET evidence of systemic avidity (*n* = 9) (%)	2 (22)	—	—	—
CSF pleocytosis (*n* = 9) (%)	2 (22)	—	—	—
Systemic inflammation (CRP) (*n* = 10) (%)	6 (60)	1 (50)	2 (67)	3 (50)
Exposure to vascular risk factors^a^ (%)	5 (45)	1 (50)	3 (100)	1 (17)
Prior evidence of immune dysfunction^b^ (%)	4 (36)	2 (100)	0	2 (33)
				
Multifocal intracranial stenosis on imaging *n* (%)	4 (36)	2 (100)	1(33)	1 (17)
Antiviral treatment *n* (%)^d^	4 (36)	2 (100)	0	3 (50)
Immunosuppressive therapy *n* (%)	8 (73)	2 (100)	2 (67)	4 (67)
Standard stroke preventative treatment^c^ *n* (%)	9 (82)	1 (50)	3 (100)	5 (83)

*n* = 11 except for when stated.

^a^ hypertension, diabetes, hypercholesterolaemia, smoker.

^b^ including ankylosing spondylitis, long-term immunosuppressive treatment, combined immunodeficiency, hypothyroidism, Takayasu disease, lichen planus, coeliac, eczema and polymyalgia rheumatica vasculitis.

^c^ including antiplatelets (aspirin, clopidogrel, warfarin) and statins.

^d^ Treatment dosing with intravenous acyclovir (10 mg/kg twice daily for 14 days) was used for those with proven.

Herpes viral infection and a preventative dosing regime were selected with valacyclovir (500 mg BD PO) whilst intrathecal antibody synthesis assay results were awaited.

‘Inflam+’; Radiological evidence of medium–large vessel inflammation with supporting evidence of inflammation from additional testing. ‘Inflam-’; Radiological evidence of medium–large vessel inflammation with no supporting evidence of inflammation from additional testing.

**Table 2:** Clinical features, treatment and outcome

Patient No.	1	2	3	4	5	6	7	8	9	10	11
Sex	F	M	F	F	F	M	F	F	M	F	F
Ethnicity^a^	Asian	White	-	White	Asian	White	White	White	White	White	Other
Age (years)	36	36	33	50	41	53	25	48	29	50	34
Type of medium-large intracranial vasculitis	Inflam+	Inflam-	Inflam-	Inflam-	Inflam+	Inflam-	Infective	Inflam-	Inflam-	Inflam+	Infective
*Presentation*											
Stroke/TIA	Stroke’	Stroke'‘	Stroke’	Stroke'‘	Stroke’	TIA'‘	Stroke’	Stroke'‘	TIA	Stroke'‘	Stroke
Headache	No	No	No	No	No	No	Yes	No	No	No	Yes
Speech disturbance^b^	No	No	No	No	Yes	Yes	No	No	No	No	No
Focal weakness or numbness	No	No	No	Yes’	-	Yes	Yes’	Yes’	-	Yes’	Yes
Visual symptoms^c^	No	No	No	No	No	No	Yes	No	Yes	No	No
Lethargy	No	No	No	No	No	No	Yes	No	No	No	Yes
Previous TIA/Stroke	Yes	No	No	Yes	No	No	Yes	No	No	Yes	Yes
PET evidence of brain avidity	No	No	No	No	No	No	No	No	-	No	-
PET evidence of systemic avidity	No	No	No	No	Yes	No	No	No	-	Yes	-
CSF pleocytosis^d^	Yes	No	No	-	No	No	No	No	No	-	Yes
Systemic inflammation CRP^e^	Yes	No	No	Yes	No	Yes	Ye	Yes	-	Yes	No
Exposure to vascular risk factors^f^	Yes	Yes	No	Yes	Yes	No	Yes	No	No	Yes	No
Prior evidence of immune dysfunction^g^	Yes	No	No	Yes	No	No	Yes	Yes	Yes	Yes	Yes
*Treatment*											
Antivirals^h^	No	Yes	No	No	No	Yes	Yes	No	Yes	No	Yes
Immunosuppressive^i^	No	Yes	Yes	No	Yes	Yes	Yes	No	Yes	Yes	Yes
Standard stroke preventative treatment^j^	Yes	Yes	Yes	Yes	Yes	Yes	Yes	No	Yes	Yes	No
*Outcome*											
Clinical recurrence at 1 year	Yes	No	No	No	No	No	No	No	No	No	No
Improvement in intracranial vessel wall enhancement at 1 year	No	Yes	No	Yes	Yes	Yes	Yes	No	No	Yes	Yes
Improvement in cerebral perfusion at 1 year	No	No	Yes	No	Yes	Yes	Yes	No	No	Yes	Yes
Improved intracranial ToF MRA at 1 year interval*	No	No	Yes	No	No	No	Yes	No	No	No	Yes

*-* missing data.

‘left-sided.

‘’ right-sided.

^a^ as defined by United Kingdom public sector information website (gov.co.uk).

^b^ dysarthria or dysphasia.

^c^ monocular visual loss, diplopia.

^d^ yes = >5cells/mm3.

^e^ yes = >5 mmol/L.

f including hypertension, diabetes, hypercholesterolaemia, smoking.

g including ankylosing spondylitis, long-term immunosuppressive treatment, combined immunodeficiency, hypothyroidism, Takayasu disease, lichen planus, coeliac, eczema and polymyalgia rheumatica vasculitis.

h valaciclovir or aciclovir.

i including steroids, methotrexate, IVIG, prednisolone, mycophenolate, azathioprine and cyclophosphamide.

j including antiplatelets (aspirin, clopidogrel, warfarin) and statins.

*one (Patient 10) had dilatation of intracranial vessels on the baseline imaging.

Inflam+’; Radiological evidence of medium–large vessel inflammation with supporting evidence of inflammation from additional testing.

Inflam-’; Radiological evidence of medium–large vessel inflammation with no supporting evidence of inflammation from additional testing.

Patient 9 had a brain biopsy.

### Inflammatory intracranial arterial vasculopathy—infective

Two patients had herpes virus inflammatory intracranial arterial vasculopathy [one herpes simplex-2 and one varicella zoster (VZV)]. Both were women, young (26-years- and 32-years-old) and presented with stepwise symptoms (see Vignette A below). Both had evidence of immune dysfunction (seronegative arthritis and allogenic haematopoietic stem cell transplant for a prior haematological malignancy). Notably, both cases had no significant CSF pleocytosis. Both had multi-focal intracranial stenosis (Table 1).

### Vignette a: herpes simplex Type 2-inflammatory intracranial arterial vasculopathy

A 23-year-old woman presented to the stroke services after suddenly developing a clumsy left hand, facial droop and featureless headache. She was a smoker and denied illicit drug use. She had seronegative arthritis, and a history of severe primary varicella zoster (VZV) infection during pregnancy 5 years previously. Examination demonstrated an ataxic monoparesis affecting her left arm. Her NIHSS score was 2.

**Figure 1:**
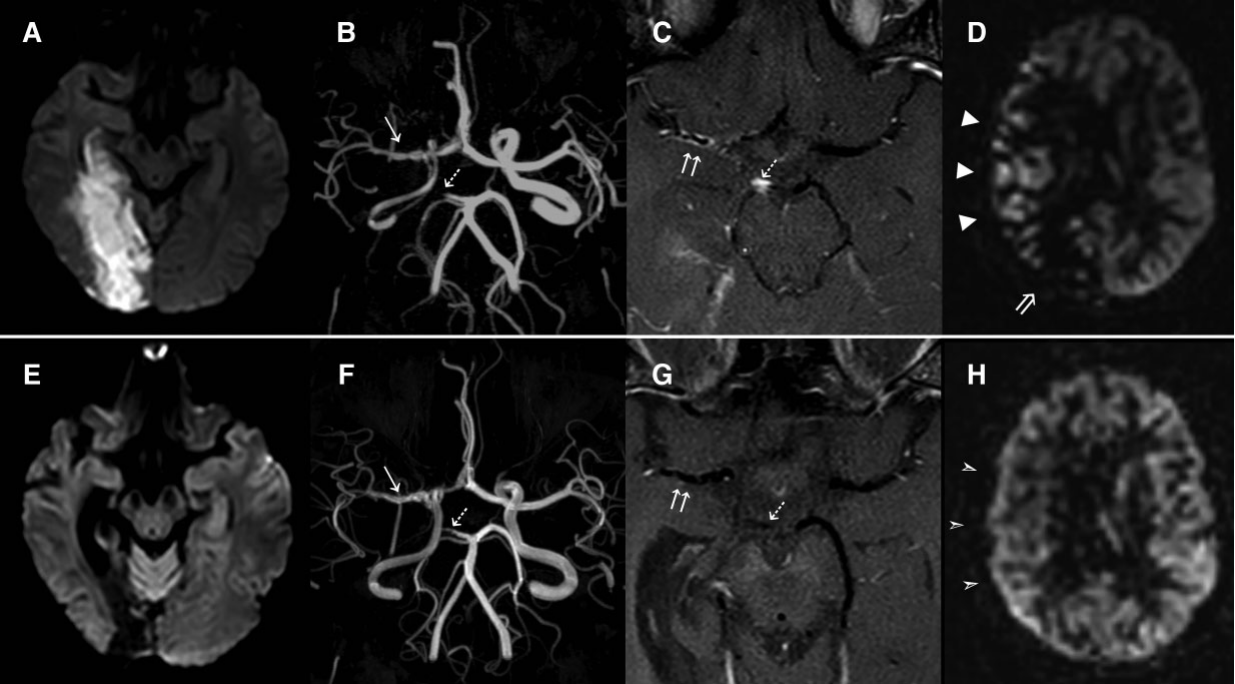
**HSV-2 inflammatory intracranial arterial vasculopathy (Patient 7);** DWI (**A**) demonstrates an acute infarct involving the right occipital and medial temporal lobes. ToF MRA (**B**) shows occlusion of the P1 segment of the right PCA (dashed arrow) and narrowing right MCA with a ‘beaded’ appearance (arrow), as well as diffuse narrowing of the intracranial right ICA. VWI (**C**) wall thickening and enhancement of the right MCA (double arrow), right P1 segment (dashed arrow) and right intracranial ICA (not shown). ASL (**D**) shows reduced perfusion in the area of infarction (arrow) and extensive ATAs over the cortical territory of the right MCA (arrowheads), indicating a relative delay in blood arrival. Follow-up imaging after treatment (**E-H**) shows the right temporo-occipital infarct maturation, with no evidence of new ischaemia on DWI (**E**). ToF MRA (**F**) demonstrates partial recanalization of the right PCA (dashed arrow), improvement in the calibre of the right MCA (arrow) and reduced narrowing of the right intracranial ICA. VWI (**G**) shows near resolution of the wall thickening and enhancement of the right MCA (double white arrows), right P1 segment (dashed arrow) and right intracranial ICA (not shown). ASL (**H**) shows normalization of perfusion in the corresponding cortical territories of the right MCA and PCA.

MRI showed an acute striatocapsular infarct and Magnetic Resonance Angiogram (MRA) revealed an occlusion of the M1 segment of the right middle cerebral artery (MCA). Baseline bloods were mostly unremarkable, including normal full blood count, urea and electrolytes, liver function, thyroid function, Vitamin B12 and folate, homocysteine levels, thrombophilia screen, fasting lipids and glucose, HIV, syphilis, hepatitis B & C, Lyme serology, autoimmune screen, 72-hour ECG recording, transesophageal echocardiogram, and thrombophilia screen. ESR (40 mm/h) and CRP (10 mg/L) were mildly elevated. On the ward she was systemically well, with improving minor symptoms and was therefore discharged on a single antiplatelet agent, lipid-lowering treatment and community therapy support. Her focal symptoms resolved at home, but she reported chronic problems with somnolence, fatigue, and a mood disorder. Seven months later, she developed a sudden onset right-sided headache, nausea and blurred vision and presented to hospital. A left homonymous hemianopia was identified on examination. Repeat MRI and MRA showed a right posterior cerebral artery (PCA) territory infarct (Fig. 1). MRA confirmed occlusion of the P1 segment of the right PCA. The right MCA had recanalised but remained narrowed and had a beaded appearance (Fig. 1). MRI vessel wall imaging demonstrated vessel enhancement in the right MCA and PCA as well as intracranial ICA. ASL showed delayed perfusion in the right MCA and PCA territories (Fig. 1). She had a LP; the opening pressure was 15 cmH_2_0. CSF examination showed four white cells/mm^3^, two red cells/mm^3^, glucose 3.2 mmoL/L (serum 4.9 mmol/L), protein 0.28 g/L, positive unmatched oligoclonal bands, and normal culture, gram stain and lactate. Notably, herpes simplex Type 2 (HSV-2) PCR was positive in her CSF (the cycle threshold cut-off was 35), and PCRs for HSV-1, VZV, cytomegalovirus, Epstein–Barr virus, human herpes virus 6 and enterovirus were negative. She was commenced on high-dose intravenous (IV) acyclovir for 7 days, 1000 mg of IV methylprednisolone for 3 days and 1 mg/kg of oral prednisolone, with suppressive 500 mg twice daily oral valacyclovir thereafter. Mycophenolate mofetil was subsequently added as a steroid-sparing agent. A repeat CSF was now negative for HSV-2 PCR, but there was evidence of monoclonal intrathecal antibody synthesis specific for HSV-2 (viral index = 7.8, normal <3), suggesting an immune response to HSV-2 in the brain. MRI showed partial recanalization of the right PCA, improvement in the right MCA and ICA calibres, reduction of vessel wall enhancement and markedly improved perfusion from the initial ASL perfusion score of 15 to a score of 29 for the right hemisphere (Fig. 1).

### Inflammatory intracranial arterial vasculopathy—radiological evidence of inflammation with additional markers (inflam+)

**Figure 2:**
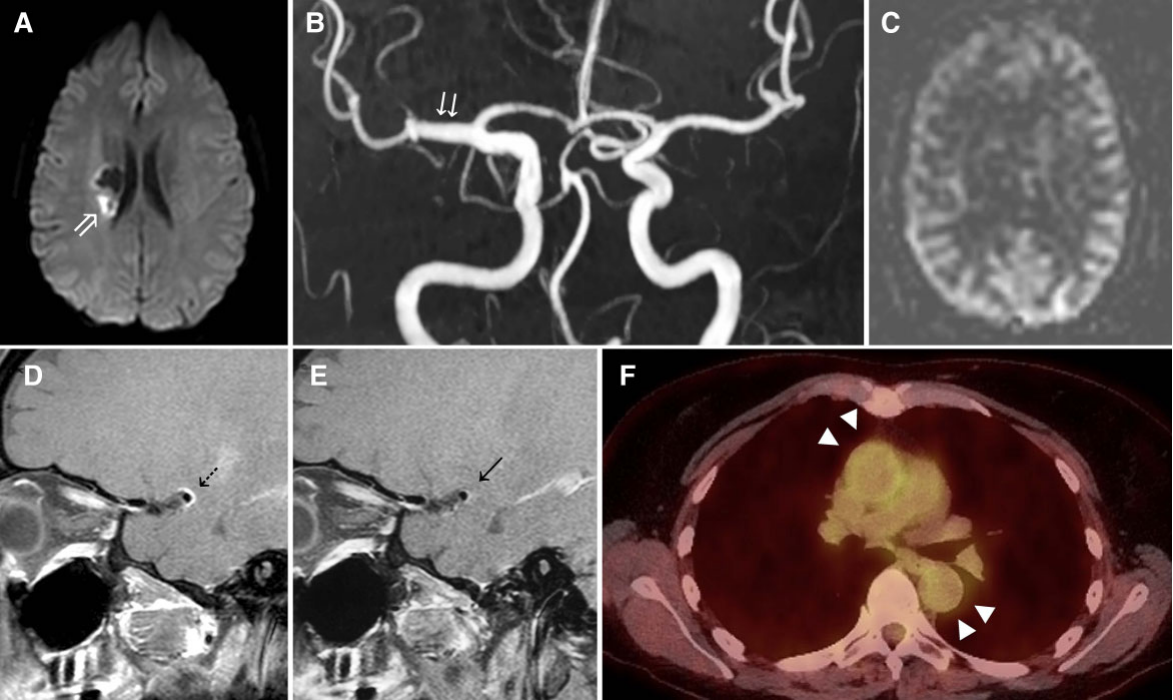
**Intracranial Takayasu (Patient 10);** Intracranial Takayasu arteritis: Axial b1000 DWI image (**A**) demonstrates an acute right striatocapsular infarct, with fusiform dilatation of the M1 segment of the right MCA on time-of-flight MRA (**B**). ASL perfusion-weighted image (**C**) shows the reduced signal intensity of labelled spins in the right peri Sylvian regions, which normalized on follow-up imaging (not shown). Axial-fused (**F**) ^18^FDG PET-CT image demonstrates concentric and increased radiotracer uptake within the wall of the ascending and proximal descending thoracic aorta (solid black arrow). Baseline sagittal VWI (**D**) shows concentric thickening and enhancement of the vessel wall of the right MCA (dotted black arrow), with interval improvement on subsequent VWI following interim treatment (**E**).

**Figure 3:**
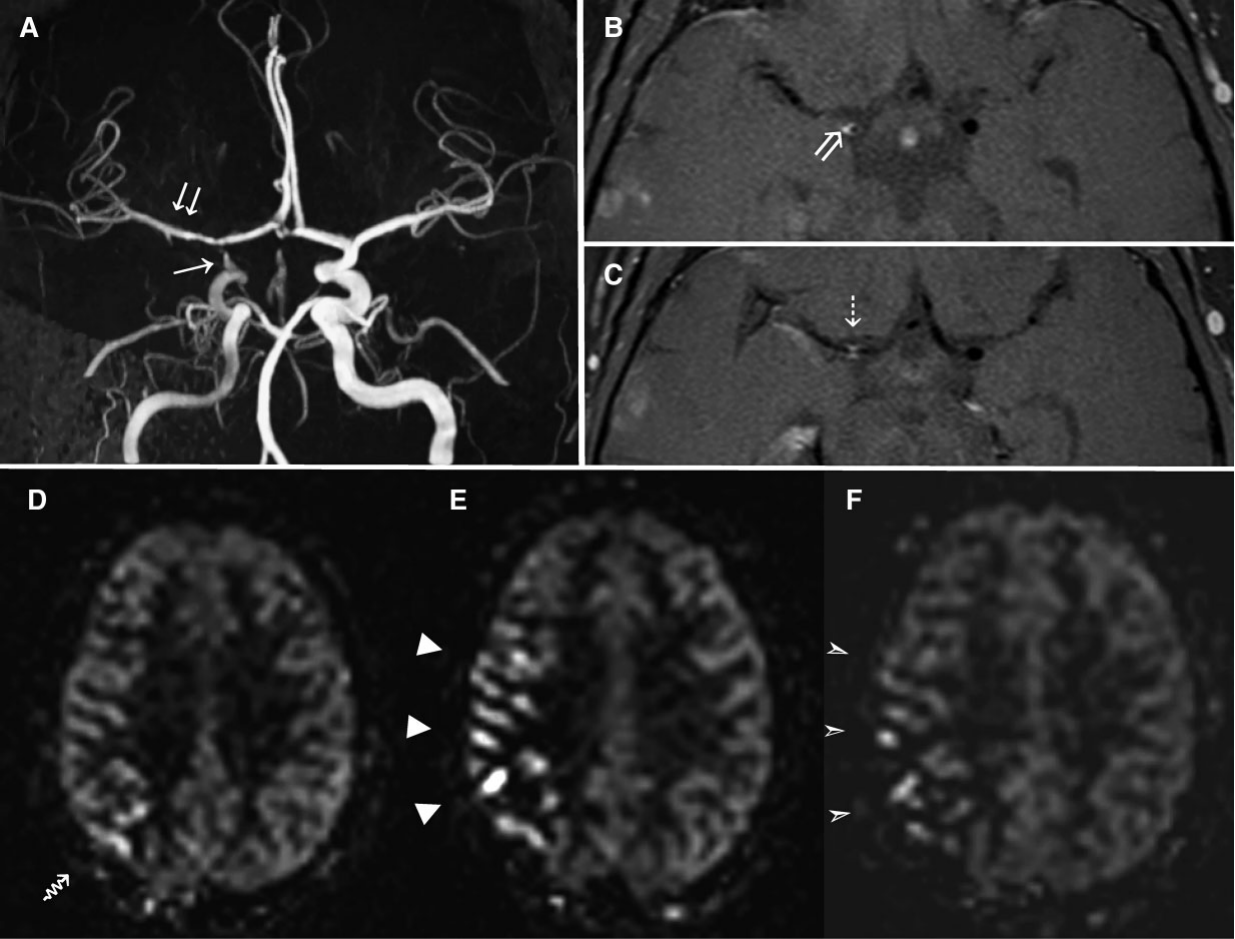
**Presumed inflammatory intracranial arterial vasculopathy ‘Inflam-’ (Patient 6)**. ToF MRA (**A**), axial VWI (**B** + **C**) and serial perfusion-weighted ASL images at supraganglionic level (**D-F**) in a 53-year-old male patient undergoing sustained immunosuppressive treatment. Baseline MRA (**A**) demonstrate irregular narrowing of the distal right ICA (arrow) and right MCA (double arrow), associated with concentric thickening and pathological enhancement of the affected vessel walls on VWI (**B** + **C**). Baseline ASL (**D**) demonstrates ATAs over the right M6 territory (arrow), with an overall ASL perfusion score of 25. ASL at 6 months (**E**) shows more extensive ATAs (arrowheads) with an overall score of 18, Subsequent ASL imaging performed at 1 year following adjustment to the patient’s treatment regime (**F**) demonstrates an associated improvement in cerebral normalisation perfusion in the right MCA territory (arrowheads), with an overall perfusion score of 22. ‘Inflam-’; Inflammatory medium–large vessel intracranial vasculitis with no supporting evidence of inflammation from additional testing

Three patients had Inflam+ indicated by an abnormal ^18^F-FDG PET scan and/or abnormal CSF. These patients had a median age of 41 years, and all three were female. Although the presence of vascular risk factor was common in this group [3 (100%)], none had a prior history of immune dysfunction. Vignette B describes a typical case, and imaging examples are presented in Fig. 2 and Fig. 3.

### Vignette b: inflam+ due to intracranial Takayasu arteritis

A 53-year-old woman presented to the stroke services with a sudden onset left-sided weakness. She has a history Takayasu arteritis and initially presented with hypertension, arthralgia and had evidence of an elevated inflammatory response. The diagnosis was made 7 months previously, following evidence of large vessel uptake on ^18^F-FDG uptake involving her ascending and descending aorta. She was managed at the time of presentation with mycophenolate and methotrexate but methotrexate had been recently interrupted because of side effects, and she was bridged with steroids. Before her admission, she had an ^18^F-FDG PET MR, which showed increased avidity in the aortic region (Fig. 2) with additional ^18^F-FDG uptake in the mesenteric and para-aortic lymph nodes. On examination, she has a left pronator drift and an MRC grade 4 +  of 5 weakness of her left upper and lower limb. She had ataxia of her affected limb and was also left-sided hyper-reflexia and a left extensor plantar response. She was found to have subclinical hypothyroidism with a TSH of 8.18 mU/L and T4 of 17.4 pmol/L and was started on levothyroxine. Full blood count, urea and electrolytes, liver function, lipid profile, HBA1C, HIV, syphilis and hepatitis B and C serology were negative or within normal limits. CSF was not performed.

The MRI of the brain showed a right, acute striatocapsular infarct corresponding to the territory of the perforating vessels, compatible with her symptoms and arising from the M1 segment of the right MCA which was not narrowed but abnormally dilated. In addition, the MRI showed an old silent cerebral infarct (Fig. 2). Dedicated vessel wall MRI demonstrated circumferential vessel wall enhancement in the right M1 segment (Fig. 2), extending into the inferior M2 segment and less marked enhancement in the supraclinoid portion of the right ICA. Active Takayasu arteritis involving the intracranial arteries was diagnosed, and she was treated with three doses of 500 mg IV methylprednisolone and subsequently recommenced on 40 mg oral prednisolone and cyclophosphamide 750 mg IV per month for 6 months. Vessel wall enhancement but not the vessel dilatation improved after treatment (Fig. 2).

### Inflammatory intracranial arterial vasculopathy—radiological evidence of inflammation with no supporting evidence from additional testing (inflam-)

Six patients had ‘Inflam-’ as indicated by normal ^18^F-FDG PET and/or CSF. These patients had a median age of 45 years (IQR: 36,53), and three (50%) were women. This group were less likely to have accompanying vascular risk 1 (17%), or prior evidence of immune dysfunction 2 (33%). Vignette C describes a typical case.

### Vignette c: inflam- and presumed inflammatory intracranial arterial vasculopathy

**Figure 4:**
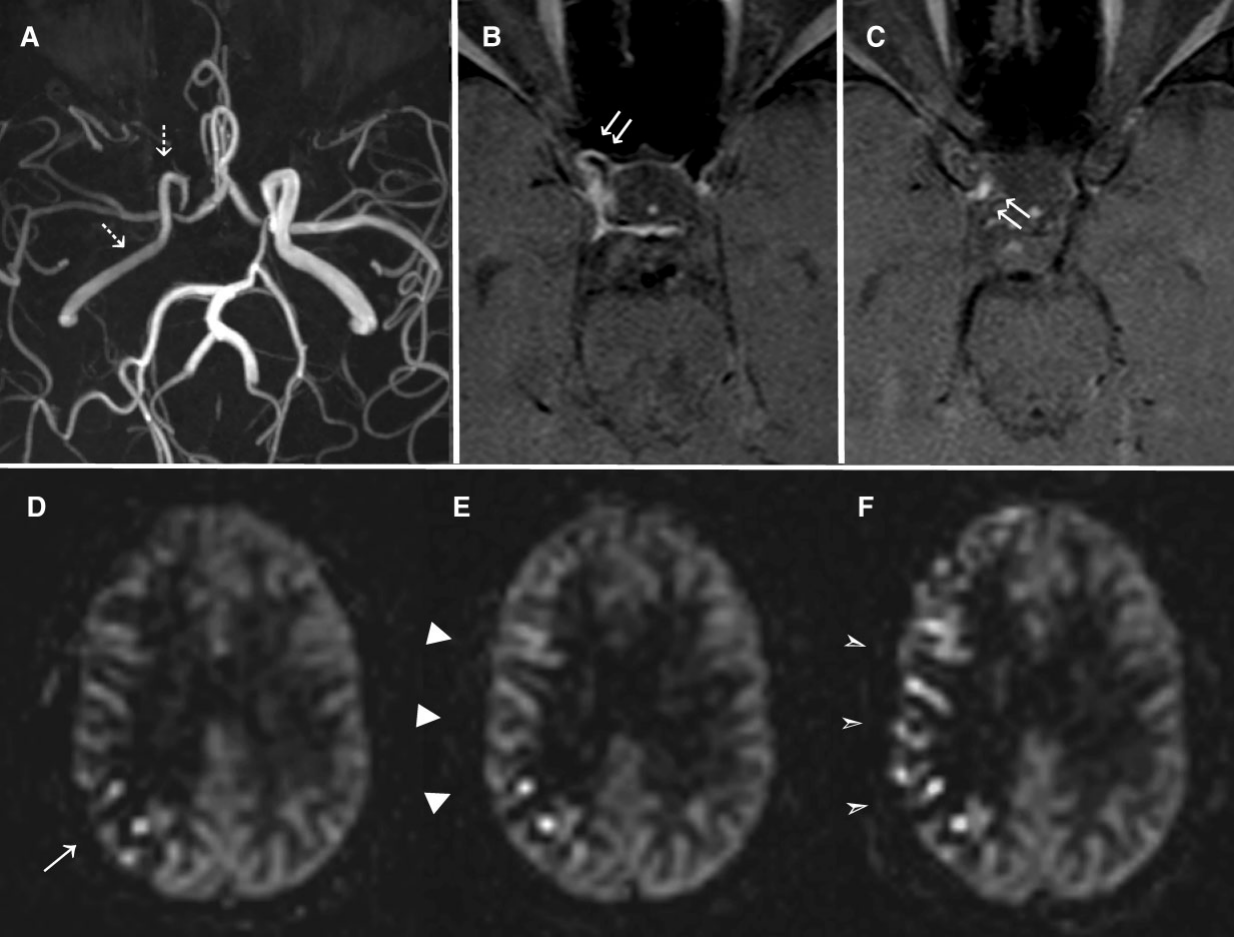
**Presumed inflammatory intracranial arterial vasculopathy ‘Inflam-’ (Patient 8);** Luminal, vessel wall and serial ASL imaging in a 48-year-old female patient without sustained treatment. Baseline ToF MRA (**A**) demonstrates tapered narrowing of the intracranial portions of the right ICA culminating in high-grade stenosis involving the right terminal ICA segment and T junction. Baseline axial VWI (**B** + **C**) demonstrates concentric vessel wall thickening and pathological enhancement of the petrous (not shown), cavernous (**B**) and supraclinoid (**C**) segments of the right ICA (double arrows). Baseline ASL (**D**) demonstrates mild ATAs over the posterior right middle cerebral artery territory, with an overall ASL perfusion score of 26, with only minimal change at 6 months (**E**). ASL performed at 1 year, following no long-term immunotherapy intervention, demonstrates worsening ATAs over the cortical territory of the right MCA, with a decline in overall perfusion score to 21 (**F**). ‘Inflam-’; Inflammatory medium–large vessel intracranial vasculitis with no supporting evidence of inflammation from additional testing.

A 48-year-old woman with a past medical history of coeliac disease, hypothyroidism, lichen planus, a high body mass index and a left cervical level 6/7 radiculopathy, presented initially to the emergency department with sudden left lower arm weakness and numbness which persisted for ∼72 h. Initial CT head, routine bloods and ECG were unremarkable. However, an MRI of her brain showed a right thalamocapsular infarct. She had a tapering narrowing of the intracranial portions of the right ICA, which had a thickened and circumferentially enhancing wall along its intracranial course, shown on her intracranial vessel wall MR, and presumed to be an inflammatory intracranial arterial vasculopathy (Fig. 4). She had ^18^F-FDG PET MRI, LP, cardiac echocardiogram and 24 h ECG monitoring which were all unremarkable. The following tests were all negative or within normal limits: thrombophilia screen, D-dimer, total homocysteine, JAK 2 mutation analysis, viral serology (including hepatitis B and C and HIV serology), ANA, double-stranded DNA, ENA, ANCA, rheumatoid factor, anti-citrullinated protein antibody, urea and electrolyte, liver function, iron, B12, folate, calcium and vitamin D. She had an MTHFR homozygous polymorphism which was not of clinical significance in the context of a normal homocysteine and serum folate levels. She did, however, have modestly elevated CRP (18 mg/L), which was noted to be elevated to a similar degree in the community. It is unclear the reason for her elevated CRP but her autoimmune co-morbidities could offer a possible explanation. Her initial antiphospholipid antibody serology was negative but she developed a transiently positive anticardiolipin antibody, and a significantly elevated Beta-2-glycoprotein-1 antibody (>100 U/L) within the first 3 months; the latter persisted for several months before antiphospholipid syndrome was diagnosed. However, this did not fully explain the clinical picture. Anticoagulation with warfarin was eventually commenced. Within the first 3 months of high dose I.V. methylprednisolone for presumed inflammatory intracranial arterial vasculopathy, but the patient declined further intervention after this. She remained free of focal symptoms. Over the course of 1 year, her right intracranial stenosis, the corresponding vessel wall enhancement and cerebral hypoperfusion of the right middle cerebral artery territory had progressed without treatment (Fig. 4).

### Impact of treatment on intracranial vascular imaging outcome

Eight of eleven patients (73%) had immunosuppressive treatment continued for more than 4 weeks. As a minimum, this included high-dose corticosteroids in all treated cases. Six received additional therapy, including methotrexate, cyclophosphamide, or mycophenolate mofetil. Treatment doses of intravenous antiviral therapy were given to those with proven herpes viral infection. Preventative oral antiviral therapy was often used alongside those taking more than one immunosuppressive treatment. The proportion with improved (reduced) wall enhancement in those treated with immunosuppressive therapy (with or without antiviral therapy) compared to the proportion of those not treated with immunosuppression with reduced wall enhancement after 1 year, was not significant [6 (86%) versus 4 (50%) (*P* = 0.201)]. We showed improvement in cerebral perfusion at 1 year using ASL sequence in patients taking immunosuppressive therapy for >4 weeks compared with those not receiving therapy [6 (100%) versus 2 (40%) *P* = 0.026]. There was no appreciable change in intracranial ToF angiogram across the treatment groups at 1 year (Table 3). Only one patient had a further clinical event. This patient was in the ‘Inflam+’ group, and at the time of the second event, the patient was not taking immunosuppressive treatment.

**Table 3:** Radiological and clinical outcome by treatment

	Improved intracranial vessel wall enhancement imaging at 1-year interval			Improved cerebral perfusion at 1-year interval			Improved intracranial ToF MRA at 1-year interval*		
	Yes *n* = 7	No *n* = 4	*P* value	Yes *n* = 6	No *n* = 5	*P* value	Yes *n* = 3	No *n* = 8	*P* value
Immunosuppressive therapy with or without antivirals *n* (%)	6 (86)	2 (50)	0.201	6 (100)	2 (40)	**0**.**026**	3 (100)	5 (63)	0.214
Immunosuppressive monotherapy *n* (%)	2 (29)	1 (25)	0.898	3 (50)	0	0.064	1 (33)	2 (25)	0.782
Immunosuppressive combined with antivirals *n* (%)	4 (57)	3 (43)	0.30	3 (50)	2 (40)	0.740	2 (67)	3 (38)	0.387

* Ten patients had stenoses and one (Patient 10) had dilatation of intracranial vessels on the baseline imaging. ToF, time of flight. Bold value indicates statistical significance.

## Discussion

We provide new evidence that non-invasive imaging of vessel wall enhancement and cerebral perfusion might be helpful in diagnosing and monitoring those with presumed inflammatory intracranial arterial vasculopathy, due to a range of underlying causes. In a case series of patients with presumed inflammatory intracranial arterial vasculopathy affecting medium–large arteries and followed up for 1 year, we used intracranial MR with ASL perfusion to aid diagnosis and management. To date, no detailed longitudinal clinical stroke series of this nature using intracranial vessel wall MRI with ASL exist. Our cohort describes a heterogenous aetiology, including infectious causes, systemic vasculitis involving intracranial arteries and presumed inflammatory intracranial arterial vasculopathy limited to the brain. Moreover, vessel wall MRI with ASL limited the need to progress to brain biopsy and strengthened our ability to monitor disease activity and adapt our management strategy once treatment was initiated.

### Treatable aetiologies

We describe novel imaging findings in two rare causes of presumed inflammatory intracranial arterial vasculopathy, namely HSV-2 and Takayasu arteritis. HSV-2 is a recognized cause of meningitis but rarely causes stroke. First, we demonstrated a progressive inflammatory intracranial arterial vasculopathy in the context of both HSV-2 DNA CSF detection and evidence of HSV-2 intra-thecal antibody production with clinical and radiological improvement following specific antiviral and anti-inflammatory therapy.^20^ HSV-2 belongs to the alpha-herpes virus family, which also includes VZV. VZV has the strongest relationship with stroke among all the herpesviruses.^21^ A short term increased risk of stroke following reactivation of an ophthalmic zoster is the most convincing; the latter implicates the trigeminal ganglion.^22^ Although HSV-2 classically establishes latency in the sacral ganglion, it can also occur in the trigeminal ganglion.^23^ The alignment of the clinical and radiological features of the case makes it biologically plausible that HSV-2 was causal, rather than a bystander.

The patient with Takayasu arteritis was a woman aged 53 years old, who had a confirmed stroke following a brief interruption of her immunosuppressive treatment for Takayasu arteritis. Given her age, the presence of a vascular risk factor, and the association with Takayasu and elevated lipoprotein,^24^ it was initially thought possible that the stroke was caused by atherosclerosis. Stroke is rare in Takayasu arteritis and is often attributed to premature atherosclerotic disease.^25,26^ However, in our case, evidence of focal inflammatory intracranial arterial vasculopathy affecting the middle cerebral artery on intracranial vessel wall MRI with a corresponding striatocapsular infarct and hypoperfusion of the respective hemisphere on ASL, indicated an active inflammatory vasculopathy and redirected her management to intensive immunomodulatory therapy with effect.

### Current diagnostic role for MRI vessel wall imaging

The current recommendations for investigating medium–large vessel intracranial vasculitis manifesting with stroke have considerable limitations. First, while MRA, CTA and US imaging (including transcranial doppler) might allow diagnosis of luminal narrowing and non-invasive monitoring of arterial injury, they remain limited in their ability to confirm inflammation and monitor disease activity.^7^ Intracranial vessel wall MRI looks beyond the lumen at the vessels, and thus provides added value.^15,16^ It also helps distinguish tramline or circumferential vessel wall enhancement (depending on the orientation of the vessel to the imaging plane), which is typical for non-atherosclerotic inflammatory vasculopathy from atherosclerosis where the enhancement is eccentric.^15,16^ It is important to note that all our patients in Category 3 (radiological evidence of inflammation with no supporting evidence from additional testing) demonstrated circumferential or tramline enhancement and not an enhancement pattern characteristic of an active arteriosclerotic plaque. Although gadolinium-based perfusion could theoretically be used to assess the cerebral haemodynamics instead of ASL, it requires more post-processing, including a deconvolution analysis and selection of arterial input function, which in the presence of intracranial stenosis can lead to less reliable results due to problems with delay and dispersion.^27^ While ^18^F-FDG PET CT/MRI has value in demonstrating inflammation systemically in the large vessel vasculitides; it is insensitive for detecting focal inflammatory intracranial arterial vasculopathy affecting the medium–large arteries, as shown in our series. Novel, more specific and sensitive PET tracers may prove helpful in the future, but currently, this remains a limitation. Intracranial vessel wall MR with black blood sequences and ASL is a recent technique which holds promise in the diagnosis and differential diagnosis of inflammatory intracranial vasculopathy.^16^ Here we demonstrated its potential in monitoring disease activity.

Second, the rapid flow of patients through the stroke pathway and the requirement for immediate use of antiplatelet therapy limits obtaining rapid investigations such as an LP or brain biopsy. A high index of suspicion is required to justify these investigations. Only 56% of our cohort had any evidence of systemic inflammation, showing that evidence on routine investigations for an underlying inflammatory pathology is often lacking in confirmed cases. Moreover, in our series even when an LP is performed, we found that just under one-third have evidence of pleocytosis and thus normal cerebrospinal fluid does not exclude an inflammatory intracranial arterial vasculopathy.

Third, while brain biopsy demonstrating inflammatory infiltrates and fibrinoid necrosis of vessel walls remains the gold standard for diagnosing intracranial arterial vasculitis, there are several disadvantages, including significant rates of false negatives and the invasiveness of the procedure leading to cerebral haemorrhage or infection.^3,24^ Furthermore, given the skipped or focal pattern of stenosis in medium–large vessel inflammatory intracranial vasculopathy, there is a possibility of non-diagnostic biopsies, with a limited sensitivity of only 53–63%.^28^ We have shown in the current series that the use of intracranial vessel wall MRI with ASL supports the diagnosis of presumed inflammatory intracranial arterial vasculopathy in suspected cases and potentially avoids the need for brain biopsy.

### Impact of immunosuppressive treatment

There are no randomized controlled trials to guide the treatment of cerebral vasculitis, and management is extrapolated from trials from systemic vasculitis and observational studies.^4,26,29^ Limited evidence in PCNSV suggests immunosuppressive drugs cross the blood–brain barrier and may be effective.^30^ Despite the difference between the current presumed inflammatory intracranial arterial vasculopathy patients and those with classical PCNSV, and the heterogeneous use of immunosuppressive therapy in our cohort, we were still able to observe a significant improvement in cerebral perfusion after 1 year. Importantly, those with seemingly inactive (Inflam-) medium–large vessel inflammatory intracranial arterial vasculopathy progressed without immunosuppressive therapy (Fig. 4), suggesting an urgent need for the identification of additional biomarkers. Among the two infection-related vasculopathies, antiviral agents were given but both required adjunctive immunosuppressive therapy to facilitate complete resolution. Our series has demonstrated the value of intracranial vessel wall MRI with ASL perfusion measurements in monitoring the effectiveness of immunosuppressive treatment and monitoring disease progression and remission.

### Strength and limitations

Our study had important strengths. First, we were able to utilize a novel imaging technique in a stroke pathway to establish the diagnosis of an inflammatory intracranial arterial vasculopathy affecting medium–large arteries that were otherwise delayed or could have been missed. Second, we closely monitored disease activity by performing interval intracranial vessel wall MRI with ASL, at 6 months and 1 year, providing novel data in patients presenting with acute stroke symptoms. This has advanced our diagnosis and ability to monitor disease activity in the brain among patients with presumed inflammatory intracranial arterial vasculopathy or those with biopsy-proven vasculitis. Third, we used predefined standardized radiological diagnostic criteria, with review (blinded to clinical details) by two neuroradiologists. Finally, the cases referred to our dedicated clinic were all identified following application of routine arterial imaging as standard of care for assessment of stroke and TIA by the clinical service and allows estimation of the incidence (∼4%) of such cases in a metropolitan stroke/TIA patient cohort.

Our study also has limitations. Although all patients met the radiological consensus agreement of a medium–large inflammatory intracranial arterial vasculopathy, the underlying aetiology varied, this could have underpowered our observation, especially when exploring the impact of treatment on vessel wall enhancement and degree of stenosis. A notable improvement in cerebral perfusion was observed and may have contributed to a favourable clinical outcome. However, this requires further investigation. We acknowledge that intracranial vessel wall MRI requires comparison to the gold standard histology for diagnosing intracranial vasculitis. However, access to the target regions (i.e. an inflamed medium–large artery) could prove hazardous. Alternative methodologies such as randomized clinical trials that demonstrate a beneficial effect on stroke outcome may need to be sought in parallel. The numbers we were able to include were limited by the condition's rarity and follow-up was limited to 1 year. Furthermore, due to the scope of the study, we were unable to determine false-negative results. We accept that some of our cases did not always fulfil the definition of cryptogenic stroke. However, in the ‘real world situation’, these diagnoses are rarely established in the hyperacute and acute phase of the stroke admission. Therefore, at discharge from these locations, all our cases had been labelled as cryptogenic stroke, and in these scenarios, intracranial vessel wall MRI could represent a valuable screening tool to rapidly identify those that have a ‘non-conventional’ cause for their stroke. As vessel wall MRI develops, so will understanding the interpretation of the obtained imaging. For example, experience with these sequences to date recommends that although active and symptomatic atherosclerotic plaques can enhance on vessel wall MRI, there are radiological features that can help distinguish lesions with this pathology from classical inflammatory vasculopathy. Further work to formally compare the vessel wall imaging characteristics of different causes of intracranial artery injury is necessary and is planned by our group and others.^16^

## Conclusion

In our longitudinal case series, we have demonstrated the utility of intracranial vessel wall MRI combined with ASL in the detection and monitoring of disease activity of patients with presumed inflammatory intracranial arterial vasculopathy or histologically proven intracranial vasculitis presenting with stroke symptoms, limiting the need to progress to brain biopsy in the former. This provides the necessary foundation to evaluate the burden formally and support vital trials to establish optimum therapeutic approaches to demonstrate a beneficial effect on stroke outcome.

## Supplementary Material

fcac157_Supplementary_Data

## Abbreviations

ANA =: antinuclear antibody
ANCA =: antinuclear cytoplasmic antibody
ASL =: arterial spin labelling
ATAs =: arterial transit artefacts
CRP =: C-reactive protein
CXR =: chest X-ray
DWI =: diffusion-weighted imaging
ENA =: extractable nuclear antibody
ESR =: erythrocyte sedimentary rate
FLAIR =: fluid-attenuated inversion recovery
HASU =: hyperacute stroke unit
HSV-2 =: herpes simplex Type 2
FS-TSE =: fat-suppressed turbo spin echo
ICA =: internal carotid artery
IV =: intravenous
JAK 2 =: Janus kinase 2
LP =: lumbar puncture
M1,2 =: middle cerebral artery segment 1 and 2, MCA = middle cerebral artery
MDT =: multi-disciplinary team
MRA =: magnetic resonance angiography
MRC =: Medical Research Council
MTHFR =: methylenetetrahydrofolate reductase
PCA =: posterior cerebral artery
PCNSV =: primary central nervous system vasculitis
TIA =: transient ischaemic attack
ToF =: time of flight
TSH =: thyroid stimulating hormone
UCLHs =: University College London Hospitals
VZV =: varicella zoster

## References

[1] Li L, Yiin GS, Geraghty OC, et al Incidence, outcome, risk factors, and long-term prognosis of cryptogenic transient ischaemic attack and ischaemic stroke: A population-based study. Lancet Neurol. 2015;14:903–913. 26227434 10.1016/S1474-4422(15)00132-5 PMC5714616

[2] Rice CM, Scolding NJ. The diagnosis of primary central nervous system vasculitis. Pract Neurol. 2020;20:109–114. 31649101 10.1136/practneurol-2018-002002

[3] Hajj-Ali RA, Singhal AB, Benseler S, Molloy E, Calabrese LH. Primary angiitis of the CNS. Lancet Neurol. 2011;10:561–572. 21601163 10.1016/S1474-4422(11)70081-3

[4] Salvarani C, Brown RD Jr, Christianson TJH, Huston J III, Giannini C, Hunder GG. Long-term remission, relapses and maintenance therapy in adult primary central nervous system vasculitis: A single-center 35-year experience. Autoimmun Rev. 2020;19:102497. 32062032 10.1016/j.autrev.2020.102497

[5] NICE . Stroke and transient ischaemic attack in over 16s: Diagnosis and initial management. National Institute for Health and Care Excellence; 2019. 31211538

[6] Dodd KC, Emsley HCA, Desborough MJR, Chhetri SK. Periprocedural antithrombotic management for lumbar puncture: Association of British neurologists clinical guideline. Pract Neurol. 2018;18:436–446. 30154234 10.1136/practneurol-2017-001820

[7] Chen SH, Sur S, Sedighim S, et al Utility of diagnostic cerebral angiography in the management of suspected central nervous system vasculitis. J Clin Neurosci. 2019;64:98–100. 30952556 10.1016/j.jocn.2019.03.058

[8] Deb-Chatterji M, Schuster S, Haeussler V, Gerloff C, Thomalla G, Magnus T. Primary angiitis of the central nervous system: New potential imaging techniques and biomarkers in blood and cerebrospinal fluid. Front Neurol. 2019;10:568. 31244749 10.3389/fneur.2019.00568 PMC6562270

[9] Mandell DM, Mossa-Basha M, Qiao Y, et al Intracranial vessel wall MRI: Principles and expert consensus recommendations of the American society of neuroradiology. AJNR Am J Neuroradiol. 2017;38:218–229. 27469212 10.3174/ajnr.A4893 PMC7963837

[10] Lindenholz A, van der Kolk AG, Zwanenburg JJM, Hendrikse J. The use and pitfalls of intracranial vessel wall imaging: How we do it. Radiology. 2018;286:12–28. 29261469 10.1148/radiol.2017162096

[11] Kuker W, Gaertner S, Nagele T, et al Vessel wall contrast enhancement: A diagnostic sign of cerebral vasculitis. Cerebrovasc Dis. 2008;26:23–29. 18511868 10.1159/000135649 PMC2813800

[12] Eiden S, Beck C, Venhoff N, et al High-resolution contrast-enhanced vessel wall imaging in patients with suspected cerebral vasculitis: Prospective comparison of whole-brain 3D T1 SPACE versus 2D T1 black blood MRI at 3 tesla. PLoS One. 2019;14:e0213514. 30849127 10.1371/journal.pone.0213514 PMC6407784

[13] Swartz RH, Bhuta SS, Farb RI, et al Intracranial arterial wall imaging using high-resolution 3-tesla contrast-enhanced MRI. Neurology. 2009;72:627–634. 19221296 10.1212/01.wnl.0000342470.69739.b3

[14] Obusez EC, Hui F, Hajj-Ali RA, et al High-resolution MRI vessel wall imaging: Spatial and temporal patterns of reversible cerebral vasoconstriction syndrome and central nervous system vasculitis. AJNR Am J Neuroradiol. 2014;35:1527–1532. 24722305 10.3174/ajnr.A3909 PMC7964439

[15] Mossa-Basha M, Shibata DK, Hallam DK, et al Added value of vessel wall magnetic resonance imaging for differentiation of nonocclusive intracranial vasculopathies. Stroke. 2017;48:3026–3033. 29030476 10.1161/STROKEAHA.117.018227 PMC5687293

[16] Edjlali M, Qiao Y, Boulouis G, et al Vessel wall MR imaging for the detection of intracranial inflammatory vasculopathies. Cardiovasc Diagn Ther. 2020;10:1108–1119. 32968663 10.21037/cdt-20-324 PMC7487407

[17] Haller S, Zaharchuk G, Thomas DL, Lovblad KO, Barkhof F, Golay X. Arterial spin labeling perfusion of the brain: Emerging clinical applications. Radiology. 2016;281:337–356. 27755938 10.1148/radiol.2016150789

[18] Zaharchuk G, Do HM, Marks MP, Rosenberg J, Moseley ME, Steinberg GK. Arterial spin-labeling MRI can identify the presence and intensity of collateral perfusion in patients with moyamoya disease. Stroke. 2011;42:2485–2491. 21799169 10.1161/STROKEAHA.111.616466 PMC3164217

[19] Roach BA, Donahue MJ, Davis LT, et al Interrogating the functional correlates of collateralization in patients with intracranial stenosis using multimodal hemodynamic imaging. AJNR Am J Neuroradiol. 2016;37:1132–1138. 27056428 10.3174/ajnr.A4758 PMC4907824

[20] Zis P, Stritsou P, Angelidakis P, Tavernarakis A. Herpes Simplex virus type 2 encephalitis as a cause of ischemic stroke: Case report and systematic review of the literature. J Stroke Cerebrovasc Dis. 2016;25:335–339. 26542825 10.1016/j.jstrokecerebrovasdis.2015.10.002

[21] Gilden D, Cohrs RJ, Mahalingam R, Nagel MA. Varicella zoster virus vasculopathies: Diverse clinical manifestations, laboratory features, pathogenesis, and treatment. Lancet Neurol. 2009;8:731–740. 19608099 10.1016/S1474-4422(09)70134-6 PMC2814602

[22] Langan SM, Minassian C, Smeeth L, Thomas SL. Risk of stroke following herpes zoster: A self-controlled case-series study. Clin Infect Dis. 2014;58:1497–1503. 24700656 10.1093/cid/ciu098 PMC4017889

[23] Berger JR, Houff S. Neurological complications of herpes simplex virus type 2 infection. Arch Neurol. 2008; 65:596–600. 18474734 10.1001/archneur.65.5.596

[24] Adams HP Jr . Cerebral vasculitis. Handb Clin Neurol. 2014;119:475–494. 24365314 10.1016/B978-0-7020-4086-3.00031-X

[25] Park MC, Lee SW, Park YB, Chung NS, Lee SK. Clinical characteristics and outcomes of takayasu's arteritis: Analysis of 108 patients using standardised criteria for diagnosis, activity assessment, and angiographic classification. Scand J Rheumatol. 2005;34:284–292. 16195161 10.1080/03009740510026526

[26] Mason JC . Takayasu arteritis–advances in diagnosis and management. Nat Rev Rheumatol. 2010;6:406–415. 20596053 10.1038/nrrheum.2010.82

[27] Calamante F, Willats L, Gadian DG, Connelly A. Bolus delay and dispersion in perfusion MRI: Implications for tissue predictor models in stroke. Magn Reson Med. 2006;55:1180–1185. 16598717 10.1002/mrm.20873

[28] Giannini C, Salvarani C, Hunder G, Brown RD. Primary central nervous system vasculitis: Pathology and mechanisms. Acta Neuropathol. 2012;123:759–772. 22421812 10.1007/s00401-012-0973-9

[29] Salvarani C, Pipitone N, Hunder GG. Management of primary and secondary central nervous system vasculitis. Curr Opin Rheumatol. 2016;28:21–28. 26599380 10.1097/BOR.0000000000000229

[30] de Boysson H, Parienti JJ, Arquizan C, et al Maintenance therapy is associated with better long-term outcomes in adult patients with primary angiitis of the central nervous system. Rheumatology (Oxford). 2017;56:1684–1693. 28340158 10.1093/rheumatology/kex047

