# Supplementary material for: Vessel wall magnetic resonance and arterial spin labelling imaging in the management of presumed inflammatory intracranial arterial vasculopathy
Source: Brain Commun. 2022 Jun 20;4(4):fcac157. doi: 10.1093/braincomms/fcac157 (PMC9263889; doi:10.1093/braincomms/fcac157)
Supplement: fcac157_Supplementary_Data [file fcac157_supplementary_data.docx]

**Supplement Figure 1:** Illustration of the patient management flow


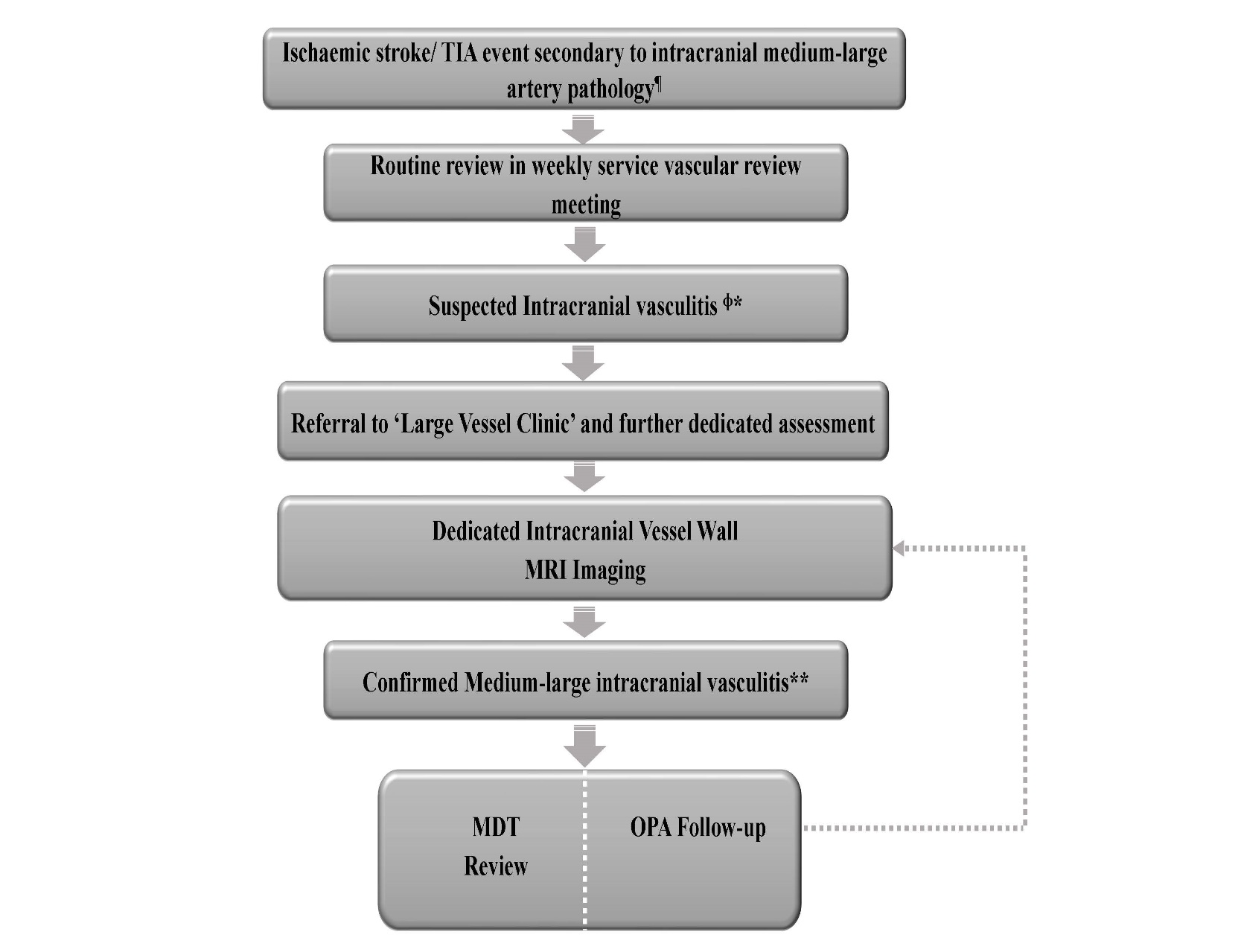


**¶** demonstration of intracranial arterial disease in territory of the stroke and discussed as routine pathway in the biweekly service MDT

**ɸ** Suspected medium-large vessel intracranial vasculitis following discussion in the MDT

*Stroke syndrome with unifocal or multifocal intracranial vessel wall narrowing or aneurysmal dilatation.

**A confirmed intracranial vasculopathy work-up included: autoimmune screen, metabolic screen, Thrombophilia screen, Haemoglobinopathy screen, chronic infection screen, CSF analysis and intrathecal

herpesvirus testing, 18F-FDG PET, +/- brain biopsy, metagenomic sequencing for unknown pathogen.
